# The Diurnal Profile of Human Basal Pain Sensitivity and Skin Sympathetic Nerve Activity: A Healthy Volunteer Study

**DOI:** 10.3389/fnins.2022.810166

**Published:** 2022-03-16

**Authors:** Ying Zhu, Ai Yan, Bin Shu, Xuehan Chen, Yupei Chen, Guangyou Duan, He Huang

**Affiliations:** Department of Anesthesiology, The Second Affiliated Hospital, Chongqing Medical University, Chongqing, China

**Keywords:** pain sensitivity, pressure pain, sympathetic nerve activity, diurnal (circadian) rhythm, cold pain

## Abstract

**Objective:**

The diurnal rhythm profile of human basal pain sensitivity and its association with sympathetic nerve activity are not fully understood. This study aimed to examine rhythmic changes in experimental pain sensitivity and skin sympathetic nerve activity in healthy volunteers.

**Methods:**

Thirty healthy volunteers were included in the study. Experimental pain sensitivity, including pressure pain threshold and tolerance, cold pain threshold (CPT) and tolerance, skin sympathetic nerve activity, and cardiovascular parameters (including heart rate, cardiac output, and peripheral vascular resistance) at six time points throughout the day (08:00, 12:00, 16:00, 20:00, 00:00, and 04:00) were sequentially measured. Circadian rhythm analysis was performed on the mean values of the different measurements and individual subjects.

**Results:**

Significant differences were found in experimental pain sensitivity, skin sympathetic nerve activity, and non-invasive cardiovascular parameters at different time points (*P* < 0.05). The minimum measured values of all four types of experimental pain sensitivity were consistently observed at 04:00. Rhythmical analysis showed that the mean values of pressure pain threshold (meta2d *P* = 0.016) and skin sympathetic nerve activity (meta2d *P* = 0.039) were significant. Significant diurnal rhythms in pain sensitivity and skin sympathetic nerve activity existed in some individuals but not in others. No significant correlation between experimental pain sensitivity and skin sympathetic nerve activity was found at any time point (*P* > 0.05).

**Conclusion:**

Significant diurnal fluctuations were observed in different pain sensitivities and skin sympathetic nerve activity. No significant correlation between experimental pain sensitivity and sympathetic excitability at different times was found; the reasons for these phenomena remain to be further studied.

**Clinical Trial Registration:**

[www.ClinicalTrials.gov], identifier [ChiCTR2000039709].

## Introduction

Pain, one of the five vital signs, is now defined as an unpleasant sensory and emotional experience associated with, or resembling that associated with, actual or potential tissue damage (Raja et al., 2020). Even with the rapid development of different analgesic drugs and analgesic methods, acute and chronic pain is still the most common problem in patients, and the treatment and relief of pain remain challenging. Human basal pain sensitivity, measured by experimental pain stimulus, is one of the main factors that affect the human body’s response to noxious stimulation and related analgesic effect (Lotsch et al., 2017; Nahman-Averbuch et al., 2019; Wen et al., 2020). Therefore, clarifying and studying the factors that affect human pain sensitivity can help predict the degree of pain and guide pain treatment.

A diurnal rhythm is a periodic or rhythmic change in the physiological or behavioral characteristics of an organism, with a periodicity of approximately 24 h, which regulates behavior, organs, and cells in living organisms (Hastings et al., 2003; Mohawk et al., 2012). The circadian system is tightly coupled with processes that control both sleep and metabolism (Huang et al., 2011). Combining the literature on pain rhythm, we found that a variety of pain has obvious diurnal qualities, but different types of pain have different rhythm characteristics (Junker and Wirz, 2010). For example, the pain peak of rheumatoid arthritis and osteoarthritis occurs in the morning, but neuropathic pain peaks usually occur during the evening time (Gilron and Ghasemlou, 2014). The peak for migraine is often in the morning or at noon, but the peak for cluster headache is mostly at midnight (Burish et al., 2019). However, although the diurnal rhythms of different types of pain have been demonstrated, research on the diurnal rhythms of basal pain sensitivity remains lacking. And the mechanism of diurnal rhythm in pain remains unclear. A recent report has simply summarized the interaction between chronic pain and circadian rhythm (Warfield et al., 2021), including the effect of interrupting circadian clock gene expression after injury and its role in the induction and maintenance of chronic pain, and the effect of chronic pain on peripheral and central circadian rhythm.

Furthermore, some studies have shown that the autonomic nervous system also has a diurnal rhythm in its regulatory effect on the body. Previous studies in a middle-aged population have used heart rate variability to measure the function of autonomic nerves, suggesting that the sympathetic nervous system of the heart is the most excitable in the afternoon, while the parasympathetic nervous system is the most excitable at night (Li et al., 2011). The autonomic nervous system is pathophysiologically related to both acute and chronic pain (Di Franco et al., 2009; Doroshenko et al., 2021). When a stress response occurs, extensive sympathetic activation can temporarily increase the nociceptive threshold through a combination of neurological and endocrine effects (Doroshenko et al., 2021). In clinical practice, sympathetic nerve block has been used for the treatment of various types of pain (visceral, vascular, and neuropathic pain); for example, abdominal ganglion block can effectively improve pain (Alexander, 1994; Jang et al., 2015; Baek and Erdek, 2019; Salman et al., 2021). Additionally, one report suggests that perioperative beta-blocker use is associated with reduced prescription opioid use at 30 days after surgery (Starr et al., 2019), indicating that sympathetic nerve activity may be associated with pain intensity. Therefore, we speculate that the diurnal rhythm of the autonomic nervous system may be related to the diurnal rhythm of pain sensitivity.

In recent years, a new method (neuECG) has been used to non-invasively record electrocardiograms (ECGs) to estimate human skin sympathetic nerve activity (Doytchinova et al., 2017). Therefore, combined with the established methods for measuring human basal pain sensitivity, this technique allows us to assess diurnal changes in sympathetic responsiveness and to analyze its association with basal pain sensitivity. Based on the above information, this study aimed to recruit volunteers to test experimental pain sensitivity, skin sympathetic nerve activity, and basic vital signs, and to explore the diurnal rhythm changes and potential associations between human pain sensitivity and sympathetic nerve activity.

## Materials and Methods

### Subjects

This study was designed as a healthy volunteer trial to determine the diurnal rhythm of basal pain sensitivity, skin sympathetic nerve activity, and their association. The study protocol was approved by the Medical Ethics Committee of the Second Affiliated Hospital of Chongqing Medical University (Ethics Committee Approval Document No.: 2020-97-1) and registered in the China Clinical Trial Center (Registration No.: ChiCTR2000039709). All subjects provided informed consent prior to the study.

From November 2020 to January 2021, 30 healthy volunteers (15 women and 15 men) from the Second Affiliated Hospital of Chongqing Medical University were recruited. Inclusion criteria were: between 18 and 35 years old, no known physical and mental diseases, able to understand and sign the informed consent and able to cooperate with the experiment, and right-handedness. Exclusion criteria were: complicating hypertension, coronary heart disease, diabetes, Parkinson’s disease and similar conditions, pacemakers/defibrillators, ventricular assist devices, and other devices installed in the heart; headache, low back pain, neuropathic pain and other acute and chronic pain; use of anti-inflammatory drugs, opioids, or related disease treatment drugs in the recent 3 months; pregnant or menstruating; inability to tolerate or cooperate with the study protocol, and heavy smoking or alcohol use.

### Study Protocol

**FIGURE 1:**
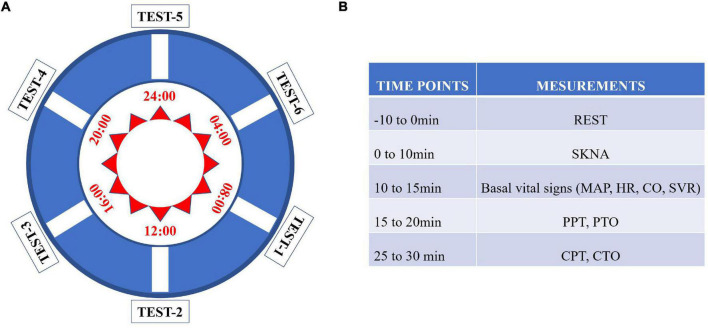
Flow chart of the study. **(A)** Each subject was tested in the chronological order from test point 1 to test point 6. **(B)** Each parameter was measured according to the listed sequence.

The test protocol was performed as shown in Figure 1. Skin sympathetic nerve activity, the basic vital signs (non-invasive cardiac displacement, blood pressure, heart rate, etc.), and pressure pain and cold pain thresholds (CPTs) were sequentially measured by the researchers at six different time points. All included subjects were asked to sleep in the same women’s or men’s dormitory and live in the same activity room and dining room in the hospital during the test procedure. During the experiment, they were required to refrain from strenuous exercise, eating spicy and stimulating food, and drinking coffee or milk tea. Light exposure was sustained from 08:00 to 22:00, while dark was sustained from 20:00 to 08:00. Subjects were given standardized instructions before the test to familiarize them with the study protocol. Each subject was required to repeat the test procedure six times a day (24 h) at 08:00, 12:00, 16:00, 20:00, 00:00, and 04:00, respectively (Figure 1), and all participants were asked to rest for 10 min before each test. All measuring equipment was placed in a quiet sampling room, and the test procedure was performed there. When the six sequential tests were performed, the subject would be invited to enter the sampling room. And when test procedure was completed during “dark” period, the subjects would be asked to stay in dark room again. The test procedures were performed in the same room, which was maintained at 25^°^C, by the same investigators.

**FIGURE 2:**
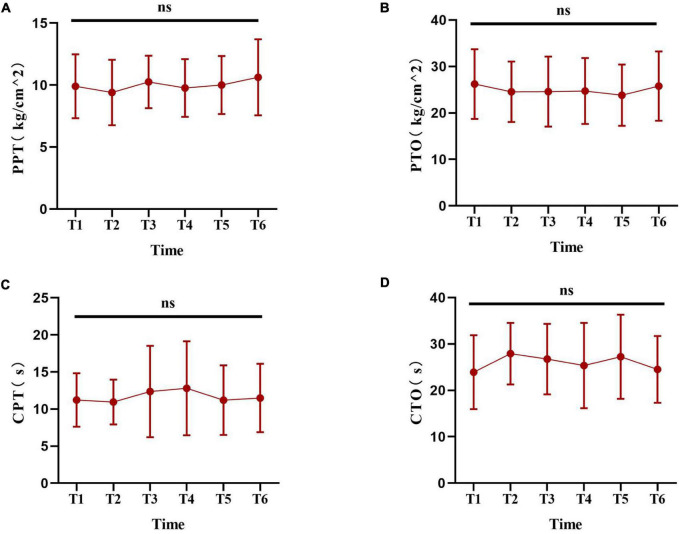
The pilot experiment based on laboratory members. **(A–D)** Show the change trend of the experimental pain thresholds (including PPT, PTO, CPT and CTO) at the six time points. Data are presented as mean ± standard deviation. PPT, pressure pain threshold; PTO, pressure pain tolerance; CPT, cold pain threshold; CTO, cold pain tolerance.

Before the experiment, a pre-experiment of pain measurement was carried out to validate the feasibility of the experimental pain measurements. In this study, six experimental pain measurements were performed within 24 h, and it was unknown whether previous sequential experimental pain measurement at any time point affected the next pain measurement. A search of the literature searching did not reveal related evidence. Thus, we designed a pilot study to perform sequential experimental pain measurements at 30 min intervals. Ten healthy laboratory members in our research group were had pain measurements taken every half an hour in 1day. The method of pain measurement was the same as that used in the experimental design. The results of the pilot study showed that no significant change was found in tests performed at 30 min intervals (Figure 2), indicating that previous sequential experimental pain measurement at any time point did not affect the next pain measurement in the current protocol. Thus, in the current protocol, the use of a 4-h interval was feasible.

### Skin Sympathetic Nerve Activity Test

The skin sympathetic nerve activity was tested using an AD Instruments device, which includes a bioelectric amplifier, PowerLab 26T, five electrocardiogram cables, and a USB connector connected to the computer. The LabChart software that matches the device is required to be installed on the computer, and the ten channels are set in the LabChart software, which was described in a previous study (Kusayama et al., 2020). This device can collect nerve ECGs and filter out the signal of the skin sympathetic nerves by setting the channels. The frequency range of adult ECGs is 0.05–150 Hz (Kligfield and Okin, 2007), and most myopotential signals are less than 100 Hz (McAuley et al., 1997). However, the frequency of the skin sympathetic nerve can range from 0 to > 2,000 Hz, and the amplitude can range from 0.5 to 100 μV (Kusayama et al., 2020); hence we could set the channel frequency to filter out the voltage of the skin sympathetic nerve. The skin is innervated by sympathetic fibers (Donadio et al., 2006), and the skin sympathetic nerve activity can assess sympathetic tone to some extent.

During the test procedure, the subjects were asked to sit in an upright position and rest calmly for 10 min. Skin sympathetic nerve activity was recorded using the device and analyzed using LabChart software. Finally, the average voltages of channels 7 and 8, after 10 min of calm rest, were recorded to reflect the skin sympathetic nerve activity (Kusayama et al., 2020).

### Pressure Pain Measurement

In this study, a hand-held electromechanical pressure meter (YISIDA-DS2; Hong Kong, China) was used according to the established measurement protocol reported in a previous study (Duan et al., 2014). The measured site was the brachioradialis lateralis region of the elbow joint of the left forearm. Six adjacent markers were made on the skin in this area to ensure that the researchers could repeat the tenderness threshold measurements using the same measurement points. A soft pad was placed under the left arm, and the pressure meter was vertically placed in the marked spot, and the pressure was increased at a rate of approximately 3.0 kg/cm^2^/s, starting at 0 kg/cm^2^. To avoid tissue damage, the maximum force was no more than 50 kg/cm^2^. The subjects were asked to say “pain” as they began to feel it during the stimulation, and the researcher recorded the pressure value displayed on the LCD screen as the pressure pain threshold (PPT). When the subjects could not tolerate the pain, they said “stop,” and the pressure value at this time was recorded as the pressure pain tolerance (PTO). During the test, the subjects were asked to turn their heads to the other side of the test limb so that they could not see the recorded value.

### Cold Pain Measurement

The subjects’ CPT was measured using a container filled with ice water maintained at 4^°^C (Klatzkin et al., 2010). At the beginning of the test, subjects were instructed to immerse their right hand vertically into the ice water container until it reached the wrist line, without touching the sides and bottom of the container, and keeping it still to maintain the same immersion status for all subjects. To avoid possible damage, the maximum time in ice water was limited to 120 s. When their hands began to feel pain, the subjects were asked to say “pain,” and the time was recorded as the CPT. When the subjects could not tolerate it, they took out their hands, and the time was recorded as the cold pain tolerance (CTO).

### Other Characteristic Parameters

Demographic data were collected for all subjects, who were assessed using the Hospital Anxiety and Depression Scale (HADS) to exclude possible subjects with severe anxiety and depression or poor sleep quality. The HADS includes 14 items that assess symptoms of anxiety and depression over the past month (every item had scores of 0–3 on a scale of 0–21). The Pittsburgh Sleep Quality Index (PSQI) was used to evaluate sleep quality, time to fall asleep, sleep time, sleep efficiency, sleep disorders, hypnotic drugs, and daytime dysfunction in the past month (0–21 points).

### Statistical Analysis

In this study, continuous data are summarized as the mean (standard deviation) and qualitative data are summarized as the number of subjects. As described in a previous study (Wu et al., 2016), identifying periodic signals in time-series data is important in studying the circadian clock. The meta2d method is designed to analyze time-series datasets in human research, including individual subjects with data collected from the same person over time. Therefore, in this study, meta2d analysis was chosen to determine whether the measured values for the same person periodically and regularly change over time within a 24 h period. First, the meta2d method was used to estimate the circadian phases of the simulated periodic profiles for the mean values of different measurement parameters at the population level. The rhythmicity of the different measurement parameters for each subject was also analyzed using the meta2d method at the individual level.

Additionally, a comparative analysis was performed using SPSS software (IBM SPSS Statistics for Windows, Version 20.0. Armonk, NY: IBM Corp.). Repeated ANOVA was used to analyze the values of basic vital signs, skin sympathetic nerve activity, PPT, and CPT at different time points. *Post hoc* comparisons using LSD *t*-test were used to compare values at different time points. Spearman correlation analysis between experimental pain sensitivity and sympathetic nerve activity, as well as non-invasive cardiovascular parameters, was performed. Statistical significance was set at *P* < 0.05.

## Results

In this study, a total of 30 healthy subjects (15 women and 15 men) were included (age range: 22–34 years old). Demographic and baseline data of all subjects are shown in Table 1. None of the subjects were found to have related basic diseases, and no anxiety, depression, or sleep disorder was found. In the study, at 4 AM the subject would be awakened to complete the test and then continue to sleep. All subjects were able to successfully fall asleep following the test.

**TABLE 1:** Characteristics of included subjects.

	Male (*n* = 15)	Female (*n* = 15)	*P*
Age (year)	28.3(4.01)	24.0(1.31)	*P* = 0.001
Height (cm)	172.5(7.00)	158.3(5.02)	*P* < 0.001
Weight (kg)	68.5(9.01)	50.0(5.42)	*P* < 0.001
HADS-anxiety	3.60(2.53)	2.93(2.05)	*P* = 0.441
HADS-depression	3.40(2.72)	2.47(1.51)	*P* = 0.255
PSQI	5.40(2.77)	4.53(2.13)	*P* = 0.346
PPT (kg/cm^2^)	11.15(4.21)	8.73(2.88)	*P* = 0.078
PTO (kg/cm^2^)	24.79(5.92)	21.07(7.24)	*P* = 0.134
CPT (second)	9.43(3.68)	11.70(4.76)	*P* = 0.156
CPO (second)	28.59(15.53)	35.36(20.03)	*P* = 0.326
MAP (mmHg)	92.73(9.05)	84.67(10.81)	*P* = 0.035
HR (bpm)	78.67(6.63)	82.53(10.01)	*P* = 0.223
SVR (dyn*s/cm^5^)	1,029.33(205.96)	900.67(132.63)	*P* = 0.052
CO (L/min)	6.51(0.80)	6.93(0.69)	*P* = 0.130

HADS, hospital anxiety and depression scale; PSQI, Pittsburgh sleep quality index; PPT, pressure pain threshold; PTO, pressure pain tolerance; CPT, cold pain threshold; CTO, cold pain tolerance; MAP, mean arterial pressure; HR, heart rate; SVR, systemic vascular resistance; CO, cardiac output; aSKNA, average skin sympathetic nerve activity. *Multiplier in units.

**FIGURE 3:**
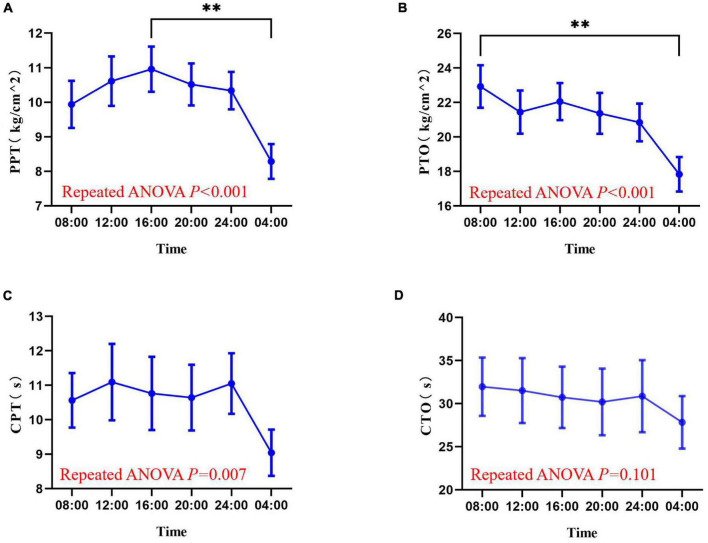
Values of pressure pain threshold **(A)**, pressure pain tolerance **(B)**, cold pain threshold **(C)**, and cold pain tolerance **(D)** at six time points in a day (08:00, 12:00, 16:00, 20:00, 00:00, 04:00). Data are represented as mean value and standard error. ^∗∗^*P* < 0.01. PPT, pressure pain threshold; PTO, pressure pain tolerance; CPT, cold pain threshold; CTO, cold pain tolerance.

The experimental pain measurements at different times are shown in Figure 3 and Supplementary Figure 1. Rhythm analysis showed that the subjects’ mean PPT had a significant rhythm (meta2d, *P* = 0.016). However, no significant rhythm was found in the mean values of PTO (meta2d, *P* = 0.354), CPT (meta2d *P* = 0.084), and CTO (meta2d *P* = 0.577). Repeated measures ANOVA showed that there were significant differences in PPT (*F* = 9.745, *P* < 0.001), PTO (*F* = 10.6145, *P* < 0.001), and CPT (*F* = 4.093, *P* = 0.007) at different time points, but no significant differences in CTO (*F* = 2.110, *P* = 0.101) at different time points. At the same time, across the six time points, each pain index had its own peak and trough values. The minimum average PPT was at 04:00 and the maximum at 16:00 [8.287 (2.7634) vs. 10.960 (3.5975) kg/cm^2^, *P* < 0.01]. The minimum average value of PTO was at 04:00 and maximum at 08:00 [17.840 (5.5037) vs. 22.930 (6.7692) kg/cm^2^, *P* < 0.01]. The minimum average CPT was at 04:00 and maximum at 12:00 [9.0430 (3.67695) vs. 11.0950 (6.06955) s, *P* = 0.119].

**FIGURE 4:**
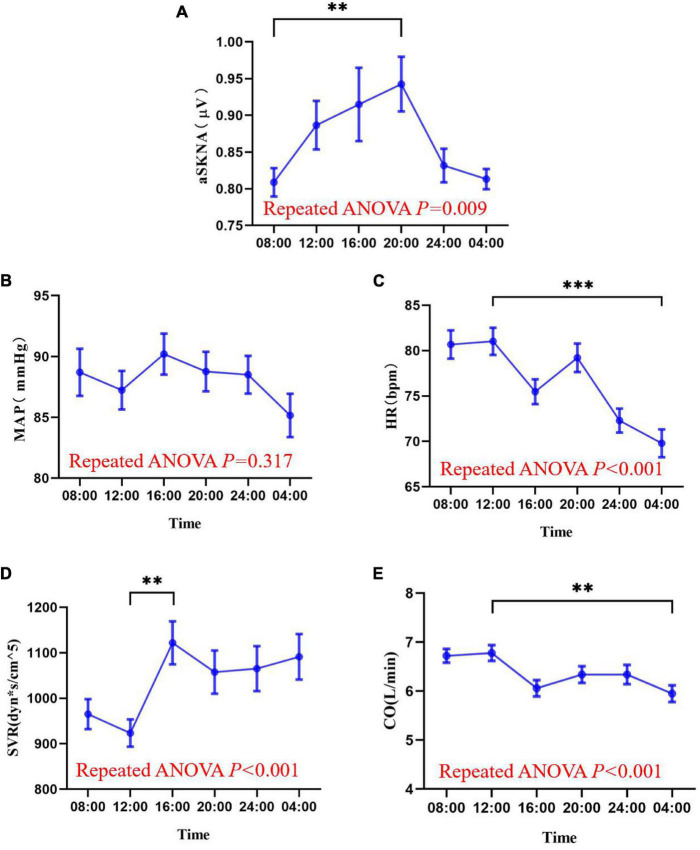
The mean values of average skin sympathetic nerve activity **(A)**, mean arterial pressure **(B)**, heart rate **(C)**, peripheral vascular resistance **(D)**, and cardiac output **(E)** at six time points of the day (08:00, 12:00, 16:00, 20:00, 00:00, 04:00). The data are shown as the mean and standard error. ^∗∗^*P* < 0.01; ^∗∗∗^*P* < 0.001. Askna, average skin sympathetic nerve activity; MAP, mean arterial pressure; HR, heart rate; SVR, systemic vascular resistance; CO, cardiac output.

Measurements of skin sympathetic nerve activity and cardiovascular parameters at six different time points are shown in Figure 4 and Supplementary Figure 2. Rhythmic analysis showed the subjects’ mean average skin sympathetic nerve activity (aSKNA) rhythms had significant rhythmicity (meta2d *P* = 0.039). The mean values of mean arterial pressure (MAP) (meta2d *P* = 0.456), heart rate (HR) (meta2d *P* = 0.256), systemic vascular resistance SVR (meta2d *P* = 0.198), and cardiac output (CO) (meta2d *P* = 0.265) showed no significant rhythmicity. Repeated ANOVA showed that there were significant differences in aSKNA (*F* = 3.976, *P* = 0.009), HR (*F* = 21.234, *P* < 0.001), SVR (*F* = 6.886, *P* < 0.001), and CO (*F* = 8.009, *P* < 0.001) at different time points. However, there was no significant difference in MAP (*F* = 1.190, *P* = 0.317) at different time points.

The minimum mean value of the average skin sympathetic nerve activity was at 08:00 and maximum at 20:00 [0.8087 (0.10670) vs. 0.9426 (0.20395) μV, *P* < 0.01]. The minimum mean value of SVR was at 12:00 and maximum at 16:00 [923.60 (164.324) vs. 1,121.70 (259.213) dyn^∗^s/cm^5^, *P* < 0.01]. The minimum mean CO was at 04:00 and maximum at 12:00 [5.950 (0.9417) vs. 6.777 (0.8697) L/min, *P* = 0.001], and HR was consistent with it, with a minimum at 04:00 and maximum at 12:00 [69.80 (8.487) vs. 81.03 (8.261) bpm, *P* < 0.001].

**FIGURE 5:**
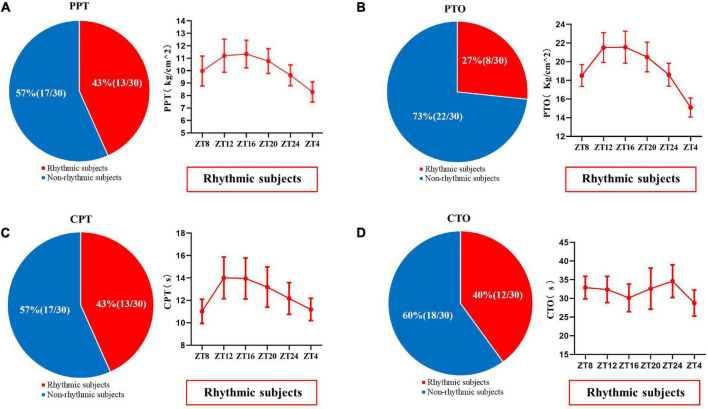
The proportion of PPT **(A)**, PTO **(B)**, CPT **(C)**, and CTO **(D)** in subjects with or without significant periodic signal. Red shows the population with significant periodic signal, and blue shows the population without significant periodic signal. The change trend of the measured values with significant periodic signal is shown. Values are presented as mean ± standard error. PPT, pressure pain threshold; PTO, pressure pain tolerance; CPT, cold pain threshold; CTO, cold pain tolerance.

**FIGURE 6:**
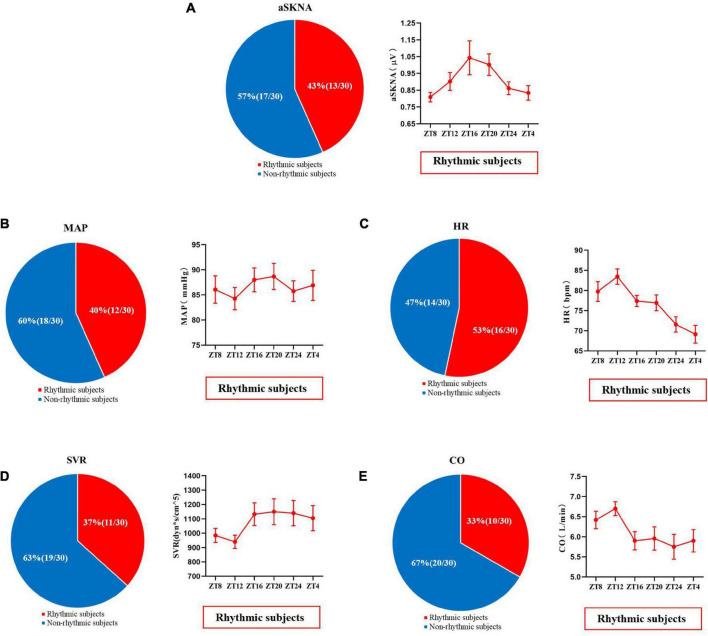
The proportion of skin sympathetic nerve voltage **(A)**, mean arterial pressure **(B)**, heart rate **(C)**, peripheral vascular resistance **(D)**, and cardiac output **(E)** in subjects with or without significant periodic signal. Red represents the population with significant periodic signal, and blue represents the population without significant periodic signal. The change trend of the measured values with significant periodic signal is shown. Values are presented as mean ± standard error. aSKNA, average skin sympathetic nerve activity; MAP, mean arterial pressure; HR, heart rate; SVR, systemic vascular resistance; CO, cardiac output.

In addition, individual rhythm analysis was used to analyze the diurnal rhythm of pain and skin sympathetic nerve activity in each subject. When *P*-value less than 0.05 in meta2d analysis for a measurement parameter, this subject would be grouped into “rhythmic subjects” for this measurement parameter, while other subjects would be grouped into “non-rhythmic subjects.” The results are shown in Figures 5, 6. In the pressure pain test, 43% (13/30) of the subjects’ PPT and 27% (8/30) of the subjects’ PTO had a significant periodic signal. In the cold pain test, 43% (13/30) of the subjects’ CPT and 40% (12/30) of the subjects’ CTO had a significant periodic signal. The trends of subjects with significant periodic signals are shown in Figure 5.

As shown in Figure 6, 43% (13/30) of the subjects showed significant changes in the diurnal rhythm of skin sympathetic nerve activity, and the average skin sympathetic nerve activity first increased from 08:00 and then decreased from 20:00. The individual rhythm analysis of other related cardiovascular indicators is shown in Figure 6.

The correlation between basal pain sensitivity, skin sympathetic nerve activity, and cardiovascular indicators is shown in Table 2. There was no significant correlation between basal pain sensitivity and sympathetic nerve activity at any of the time points. MAP showed a significant positive correlation with PPT at three different time points.

**TABLE 2:** Correlation between basal pain sensitivity and skin sympathetic nerve activity and cardiovascular index.

	PPT	*P*	PTO	*P*	CPT	*P*	CTO	*P*
aSKNA1	0.139	0.463	–0.050	0.793	0.103	0.589	0.102	0.606
aSKNA2	0.221	0.240	–0.061	0.749	–0.152	0.422	–0.093	0.638
aSKNA3	–0.072	0.706	–0.056	0.768	–0.056	0.768	–0.077	0.698
aSKNA4	–0.062	0.745	–0.020	0.917	–0.223	0.235	–0.181	0.358
aSKNA5	0.097	0.609	0.082	0.666	0.294	0.114	0.012	0.950
aSKNA6	–0.032	0.867	–0.184	0.331	–0.061	0.750	–0.210	0.284
HR1	–0.176	0.351	–0.060	0.753	0.115	0.546	–0.054	0.179
HR2	–0.191	0.313	–0.230	0.150	0.280	0.134	0.231	0.236
HR3	–0.207	0.271	–0.283	0.130	–0.181	0.337	0.110	0.578
HR4	–0.098	0.607	–0.141	0.459	–0.056	0.769	–0.013	0.947
HR5	–0.020	0.917	–0.060	0.753	–0.100	0.599	0.089	0.653
HR6	–0.07	0.714	–0.074	0.698	–0.061	0.750	0.132	0.502
CO1	–0.404	0.027	–0.412	0.024	–0.147	0.438	–0.261	0.179
CO2	–0.113	0.553	–0.005	0.979	0.268	0.152	0.313	0.105
CO3	0.077	0.686	0.208	0.270	–0.009	0.962	0.153	0.436
CO4	–0.449	0.013	–0.243	0.196	–0.199	0.291	–0.088	0.658
CO5	–0.246	0.190	–0.116	0.541	–0.243	0.196	–0.103	0.603
CO6	0.007	0.970	–0.009	0.963	–0.085	0.654	0.016	0.935
SVR1	0.431	0.017	0.253	0.177	0.084	0.661	0.021	0.916
SVR2	0.224	0.234	0.051	0.790	–0.127	0.504	–0.209	0.287
SVR3	0.164	0.387	–0.033	0.863	–0.005	0.978	–0.169	0.390
SVR4	0.584	0.001	0.337	0.069	0.154	0.417	0.045	0.818
SVR5	0.302	0.105	0.143	0.452	0.339	0.067	0.155	0.430
SVR6	0.287	0.124	0.235	0.212	0.211	0.262	0.033	0.869
MAP1	0.399	0.029	0.237	0.208	–0.011	0.953	–0.009	0.964
MAP2	0.177	0.350	0.114	0.550	0.047	0.804	–0.099	0.615
MAP3	0.338	0.068	0.248	0.186	0.016	0.934	–0.086	0.665
MAP4	0.454	0.012	0.125	0.509	0.059	0.755	–0.013	0.947
MAP5	0.211	0.263	0.116	0.541	0.033	0.861	0.030	0.880
MAP6	0.505	0.004	0.410	0.025	0.230	0.221	0.078	0.692

*PPT, pressure pain threshold; PTO, pressure pain tolerance; CPT, cold pain threshold; CTO, cold pain tolerance; MAP, mean arterial pressure; HR, heart rate; SVR, systemic vascular resistance; CO, cardiac output; aSKNA, average skin sympathetic nerve activity.*

## Discussion

In this study, it was demonstrated that the mean values of mechanical pain threshold and skin sympathetic nerve reactivity had significant diurnal rhythms. However, the individual rhythm analysis showed that not all subjects had significant diurnal rhythms, and there were differences among individuals with different physiological indexes. In addition, there was no significant correlation between the experimental pain threshold and the skin sympathetic nerve reactivity at different time points.

Regarding the diurnal rhythm of pain threshold, previous studies on the diurnal rhythm of pain only focused on pain intensity at several specific time points (Koch and Raschka, 2004). To the best of our knowledge, this study is the first to specifically analyze the rhythmicity of human basal pain sensitivity through rhythmicity analysis and to compare basal pain sensitivity at different time points. The results of ANOVA analysis showed that experimental pain sensitivity does differ significantly at different time points over 24 h. Both pressure and CPTs were lowest at 4 a.m., which is consistent with previous studies that showed that pain was more intense at night (Toporikova et al., 2017). However, in the met2d analysis, we found that in the included population, only the mean value of the PPT showed significant periodic signals. Cold pain may be affected by many factors, such as the existing rhythm of body temperature in the population (Refinetti, 2010). This may interfere with the rhythm of subjects’ perception and tolerance to cold stimuli. Meanwhile, one previous study has shown that there is no obvious rhythm change in either the perception of thermal or cold stimuli or the pain sensation generated by thermal stimulus (Strian et al., 1989). Nevertheless, the findings of this study may be helpful for clinical pain prediction and analgesia. For example, analgesia pre-administration or intensification prior to the peak of pain can be applied during clinical pain treatment. Therefore, based on the diurnal rhythm characteristics of pain sensitivity, reasonable and individualized time prescriptions be expected to optimize the analgesic strategy.

Another important indicator found in this study was the skin sympathetic nerve activity. We found that its mean values also have obvious rhythmic fluctuations. After 08:00, the skin sympathetic nervous activity showed rising trends, and after 20:00, it showed a trend of gradual decline. This is consistent with the phenomenon that the parasympathetic nerve often predominates during the night (Li et al., 2011). The temporal data of skin sympathetic nerve activity have not been reported before, but some studies have confirmed that heart rate variability shows obvious rhythmic characteristics (Singh and Rabkin, 2021), which are mainly affected by sympathetic excitability. Importantly, it lines up nicely with the morning rise of cortisol, a key biological stress response system (Adam et al., 2017), which has a synergistic effect on the sympathetic nervous system. As previously reported, cortisol follows a strong diurnal rhythm: levels are high on waking, drop rapidly in subsequent few hours after the awakening surge, and then drop more slowly until reaching a nadir around bedtime (Pruessner et al., 1997). In this study, experimental data on cardiac output and heart rate also consistently showed a gradual decline from day to night. However, according to the experimental results, the rhythmic characteristics of other cardiovascular parameter mean values were not significant. By analyzing the diurnal rhythm of individual cardiovascular parameters, we found that some participants had a significant diurnal rhythm while others did not. Individual differences may be one of the reasons why the diurnal rhythm of mean cardiovascular parameters was not significant.

Through this study, we found that there are great differences in the diurnal characteristics of various physiological parameters in different individuals. Individual variation in genetic background, personal experience, and environmental factors may contribute to this difference. This may be because the diurnal rhythm of the response of different organ systems in different subjects could be affected by different environments, backgrounds, genes, etc. Eating time, sleeping habits, cardiopulmonary adaptability, smoking, drinking, and individual chronotype may affect the circadian rhythm (Montaigne et al., 2018). An individual’s circadian rhythm can also change with environmental changes, such as the effects of artificial light (Khodasevich et al., 2021). Therefore, not all subjects showed significant diurnal rhythms in various physiological parameters. Thus, the data on diurnal rhythm may not be applicable to all individuals, and it remains necessary to consider the existence of individual rhythm differences in clinical practice.

The experimental results of this study showed that there was no significant association between basal pain sensitivity and sympathetic nerve activity at different time points. A previous study has shown that pain intensity is directly related to cardiovascular sympathetic hyperactivity in patients with fibromyalgia (Zamunér et al., 2015), indicating that sympathetic nerve excitation may enhance the pathological pain response. However, at present, no study has clearly identified the association between sympathetic nerve excitation and experimental pain sensitivity under normal conditions. Our findings suggest that sympathetic nerve excitation may not affect subjects’ immediate pain sensitivity. In contrast, previous studies found that in healthy humans, stimulation of vagal afferents could reduce experimental pain perception (Sedan et al., 2005; Busch et al., 2013). It was also found that lower parasympathetic activity was associated with higher pain sensitivity in fibromyalgia patients and chemotherapy-induced neuropathy patients (Reyes del Paso et al., 2011; Nahman-Averbuch et al., 2016). Therefore, whether basal pain sensitivity is more likely to be related to vagal nerve excitability deserves further investigation. In addition, skin sympathetic nerve activity may not be applicable to reflect subjects’ overall sympathetic activity; thus, the correlation between basal pain sensitivity and sympathetic nerve activity needs to be further studied. Because no clear association with the skin sympathetic nerve tone was observed in this study, it is important to consider other aspects which influence the diurnal rhythm of pain. Based on the definition of pain, pain sensitivity depends on an emotional experience. Mood and alertness are known to change over 24 h, and one previous study demonstrated that alertness could affect pain sensitivity (Alexandre et al., 2017). Because the level of alertness fluctuates in a circadian manner, with the lowest levels in the early morning and the highest levels during daytime (Lok et al., 2019), this might affect the diurnal rhythms of pain. In addition, a recent article suggests that the diurnal rhythms of pain are the complex result of distributed rhythms across the entire pain system, particularly those of the descending pain-regulating system and the systems that interact with it, including opioids and the endocrine and immune systems (Bumgarner et al., 2021). Thus, there is still much to be learned in studying the mechanisms underlying the diurnal rhythm of pain.

Several limitations should be considered when interpreting the results of this study. First, this study was designed as an exploratory study, and the sample size was relatively small; thus, a larger sample size study may be needed to validate the current findings. Second, the basal and demographic characteristics of the included cohort are strictly limited. For example, the age range, physical health status, and nature of work of the selected subjects are similar. Thus, in other populations, the current rhythmic results need to be further explored. Thirdly, although these rhythms in this study could be circadian and controlled by the circadian system, that would require further experiments. In addition, body temperature and vagus nerve activity were not included as measurement parameters in this study, and the rhythmic correlation between basal pain sensitivity and these factors remains to be studied in the future.

In summary, the present study describes the diurnal rhythm and time point differences in cardiovascular parameters, skin sympathetic nerve activity, and experimental pain sensitivity in healthy subjects. Diurnal rhythm fluctuations of basal pain sensitivity and skin sympathetic nerve activity do exist, which may guide the application of time-differentiated clinical analgesia and treatment of sympathetic nerve-related diseases. However, we found that significant diurnal rhythms in basal pain sensitivity and skin sympathetic nerve activity existed in some individuals but not in others. Therefore, it is necessary to clarify the characteristics of individual diurnal rhythms in time-based differentiated treatments. In addition, no significant correlation between experimental pain sensitivity and sympathetic excitability at different times was found, and the reasons for these phenomena remain to be further studied.

## Data Availability Statement

The raw data supporting the conclusions of this article will be made available by the authors, without undue reservation.

## Ethics Statement

The studies involving human participants were reviewed and approved by the Medical Ethics Committee of The Second Affiliated Hospital of Chongqing Medical University. The patients/participants provided their written informed consent to participate in this study.

## Author Contributions

YZ, AY, and XC contributed to the data collection. YZ, BS, and GD contributed to the data analysis. YZ, GD, YC, and HH contributed to the draft writing. GD and HH contributed to the draft revision. All authors gave final approval of the version to be published, and agreed to be accountable for all aspects of the work.

## Conflict of Interest

The authors declare that the research was conducted in the absence of any commercial or financial relationships that could be construed as a potential conflict of interest.

## Publisher’s Note

All claims expressed in this article are solely those of the authors and do not necessarily represent those of their affiliated organizations, or those of the publisher, the editors and the reviewers. Any product that may be evaluated in this article, or claim that may be made by its manufacturer, is not guaranteed or endorsed by the publisher.

## Supplementary Material

Supplementary Figure 1 The scatter diagrams of the values of pressure pain threshold **(A)**, pressure pain tolerance **(B)**, cold pain threshold **(C)**, and cold pain tolerance **(D)** at six time points in a day (08:00, 12:00, 16:00, 20:00, 00:00, 04:00).

Supplementary Figure 2 The scatter diagrams of the values of average skin sympathetic nerve activity **(A)**, mean arterial pressure **(B)**, heart rate **(C)**, peripheral vascular resistance **(D)**, and cardiac output **(E)** at six time points of the day (08:00, 12:00, 16:00, 20:00, 00:00, and 04:00).

## References

[B1] Adam E. K. Quinn M. E. Tavernier R. McQuillan M. T. Dahlke K. A. Gilbert K. E. (2017). Diurnal cortisol slopes and mental and physical health outcomes: a systematic review and meta-analysis. *Psychoneuroendocrinology* 83 25–41. 10.1016/j.psyneuen.2017.05.018 28578301 PMC5568897

[B2] Alexander J. P. (1994). Chemical lumbar sympathectomy in patients with severe lower limb ischaemia. *Ulster Med. J.* 63 137–143. 8650825 PMC2448769

[B3] Alexandre C. Latremoliere A. Ferreira A. Miracca G. Yamamoto M. Scammell T. E. (2017). Decreased alertness due to sleep loss increases pain sensitivity in mice. *Nat. Med.* 23 768–774. 10.1038/nm.4329 28481358 PMC5798598

[B4] Baek S. W. Erdek M. A. (2019). Time-dependent change in pain threshold following neurolytic celiac plexus block. *Pain Manag.* 9 543–550. 10.2217/pmt-2019-0021 31729281

[B5] Bumgarner J. R. Walker W. H. II Nelson R. J. (2021). Circadian rhythms and pain. *Neurosci. Biobehav. Rev.* 129 296–306. 10.1016/j.neubiorev.2021.08.004 34375675 PMC8429267

[B6] Burish M. J. Chen Z. Yoo S. H. (2019). Emerging relevance of circadian rhythms in headaches and neuropathic pain. *Acta Physiol.* 225:e13161. 10.1111/apha.13161 29969187 PMC6381824

[B7] Busch V. Zeman F. Heckel A. Menne F. Ellrich J. Eichhammer P. (2013). The effect of transcutaneous vagus nerve stimulation on pain perception–an experimental study. *Brain Stimul.* 6 202–209. 10.1016/j.brs.2012.04.006 22621941

[B8] Di Franco M. Iannuccelli C. Alessandri C. Paradiso M. Riccieri V. Libri F. (2009). Autonomic dysfunction and neuropeptide Y in fibromyalgia. *Clin. Exp. Rheumatol.* 27 S75–S78. 20074444

[B9] Donadio V. Nolano M. Provitera V. Stancanelli A. Lullo F. Liguori R. (2006). Skin sympathetic adrenergic innervation: an immunofluorescence confocal study. *Ann. Neurol.* 59 376–381. 10.1002/ana.20769 16437571

[B10] Doroshenko M. Turkot O. Horn D. B. (2021). *Sympathetic Nerve Block, StatPearls, StatPearls Publishing Copyright 2021.* Treasure Island, FL: StatPearls Publishing LLC. 32491569

[B11] Doytchinova A. Hassel J. L. Yuan Y. Lin H. Yin D. Adams D. (2017). Simultaneous noninvasive recording of skin sympathetic nerve activity and electrocardiogram. *Heart Rhythm* 14 25–33. 10.1016/j.hrthm.2016.09.019 27670627 PMC5182108

[B12] Duan G. Xiang G. Zhang X. Guo S. Zhang Y. (2014). An improvement of mechanical pain sensitivity measurement method: the smaller sized probes may detect heterogeneous sensory threshold in healthy male subjects. *Pain Med.* 15 272–280. 10.1111/pme.12245 24118900

[B13] Gilron I. Ghasemlou N. (2014). Chronobiology of chronic pain: focus on diurnal rhythmicity of neuropathic pain. *Curr. Opin. Support. Palliat. Care* 8 429–436. 10.1097/SPC.0000000000000085 25111256

[B14] Hastings M. H. Reddy A. B. Maywood E. S. (2003). A clockwork web: circadian timing in brain and periphery, in health and disease. *Nat. Rev. Neurosci.* 4 649–661. 10.1038/nrn1177 12894240

[B15] Huang W. Ramsey K. M. Marcheva B. Bass J. (2011). Circadian rhythms, sleep, and metabolism. *J. Clin. Invest.* 121 2133–2141. 21633182 10.1172/JCI46043 PMC3104765

[B16] Jang Y. H. Lee J. S. Kim S. L. Chi S. G. Lee W. J. Lee S. J. (2015). Do interventional pain management procedures during the acute phase of herpes zoster prevent postherpetic neuralgia in the elderly?: a meta-analysis of randomized controlled trials. *Ann. Dermatol.* 27 771–774. 10.5021/ad.2015.27.6.771 26719654 PMC4695437

[B17] Junker U. Wirz S. (2010). Review article: chronobiology: influence of circadian rhythms on the therapy of severe pain. *J. Oncol. Pharm. Pract.* 16 81–87. 10.1177/1078155209337665 19541762

[B18] Khodasevich D. Tsui S. Keung D. Skene D. J. Revell V. Martinez M. E. (2021). Characterizing the modern light environment and its influence on circadian rhythms. *Proc. Biol. Sci.* 288:20210721. 10.1098/rspb.2021.0721 34284625 PMC8292753

[B19] Klatzkin R. R. Mechlin B. Girdler S. S. (2010). Menstrual cycle phase does not influence gender differences in experimental pain sensitivity. *Eur. J. Pain* 14 77–82. 10.1016/j.ejpain.2009.01.002 19217329 PMC2819535

[B20] Kligfield P. Okin P. M. (2007). Prevalence and clinical implications of improper filter settings in routine electrocardiography. *Am. J. Cardiol.* 99 711–713. 10.1016/j.amjcard.2006.09.123 17317378

[B21] Koch H. J. Raschka C. (2004). Diurnal variation of pain perception in young volunteers using the tourniquet pain model. *Chronobiol. Int.* 21 171–173. 10.1081/cbi-120027989 15129831

[B22] Kusayama T. Wong J. Liu X. He W. Doytchinova A. Robinson E. A. (2020). Simultaneous noninvasive recording of electrocardiogram and skin sympathetic nerve activity (neuECG). *Nat. Protoc.* 15 1853–1877. 10.1038/s41596-020-0316-6 32313253

[B23] Li X. Shaffer M. L. Rodriguez-Colon S. He F. Wolbrette D. L. Alagona P. Jr. (2011). The circadian pattern of cardiac autonomic modulation in a middle-aged population. *Clin. Auton. Res.* 21 143–150. 10.1007/s10286-010-0112-4 21240538 PMC3093547

[B24] Lok R. van Koningsveld M. J. Gordijn M. C. M. Beersma D. G. M. Hut R. A. (2019). Daytime melatonin and light independently affect human alertness and body temperature. *J. Pineal Res.* 67:e12583. 10.1111/jpi.12583 31033013 PMC6767594

[B25] Lotsch J. Ultsch A. Kalso E. (2017). Prediction of persistent post-surgery pain by preoperative cold pain sensitivity: biomarker development with machine-learning-derived analysis. *Br. J. Anaesth.* 119 821–829. 10.1093/bja/aex236 29121286

[B26] McAuley J. H. Rothwell J. C. Marsden C. D. (1997). Frequency peaks of tremor, muscle vibration and electromyographic activity at 10 Hz, 20 Hz and 40 Hz during human finger muscle contraction may reflect rhythmicities of central neural firing. *Exp. Brain Res.* 114 525–541. 10.1007/pl00005662 9187289

[B27] Mohawk J. A. Green C. B. Takahashi J. S. (2012). Central and peripheral circadian clocks in mammals. *Annu. Rev. Neurosci.* 35 445–462. 10.1146/annurev-neuro-060909-153128 22483041 PMC3710582

[B28] Montaigne D. Marechal X. Modine T. Coisne A. Mouton S. Fayad G. (2018). Daytime variation of perioperative myocardial injury in cardiac surgery and its prevention by Rev-Erbalpha antagonism: a single-centre propensity-matched cohort study and a randomised study. *Lancet* 391 59–69. 10.1016/S0140-6736(17)32132-3 29107324

[B29] Nahman-Averbuch H. Leon E. Hunter B. M. Ding L. Hershey A. D. Powers S. W. (2019). Increased pain sensitivity but normal pain modulation in adolescents with migraine. *Pain* 160 1019–1028. 10.1097/j.pain.0000000000001477 30624343

[B30] Nahman-Averbuch H. Sprecher E. Jacob G. Yarnitsky D. (2016). The relationships between parasympathetic function and pain perception: the role of anxiety. *Pain Pract.* 16 1064–1072. 10.1111/papr.12407 26878998

[B31] Pruessner J. C. Wolf O. T. Hellhammer D. H. Buske-Kirschbaum A. von Auer K. Jobst S. (1997). Free cortisol levels after awakening: a reliable biological marker for the assessment of adrenocortical activity. *Life Sci.* 61 2539–2549. 10.1016/s0024-3205(97)01008-4 9416776

[B32] Raja S. N. Carr D. B. Cohen M. Finnerup N. B. Flor H. Gibson S. (2020). The revised international association for the study of pain definition of pain: concepts, challenges, and compromises. *Pain* 161 1976–1982. 10.1097/j.pain.0000000000001939 32694387 PMC7680716

[B33] Refinetti R. (2010). The circadian rhythm of body temperature. *Front. Biosci.* 15 564–594. 10.2741/3634 20036834

[B34] Reyes del Paso G. A. Garrido S. Pulgar A. Duschek S. (2011). Autonomic cardiovascular control and responses to experimental pain stimulation in fibromyalgia syndrome. *J. Psychosom. Res.* 70 125–134. 10.1016/j.jpsychores.2010.09.012 21262414

[B35] Salman A. S. Abbas D. N. Elrawas M. M. Kamel M. A. Mohammed A. M. Abouel Soud A. H. (2021). Postmastectomy pain syndrome after preoperative stellate ganglion block: a randomized controlled trial. *Minerva Anestesiol.* 87 786–793. 10.23736/S0375-9393.21.15112-0 33938674

[B36] Sedan O. Sprecher E. Yarnitsky D. (2005). Vagal stomach afferents inhibit somatic pain perception. *Pain* 113 354–359. 10.1016/j.pain.2004.11.012 15661444

[B37] Singh I. Rabkin S. W. (2021). Circadian variation of the QT interval and heart rate variability and their interrelationship. *J. Electrocardiol.* 65 18–27. 10.1016/j.jelectrocard.2021.01.004 33465743

[B38] Starr J. B. Backonja M. Rozet I. (2019). Beta-blocker use is associated with a reduction in opioid use 30 days after total knee arthroplasty. *Pain Physician* 22 E395–E406. 31561649

[B39] Strian F. Lautenbacher S. Galfe G. Hölzl R. (1989). Diurnal variations in pain perception and thermal sensitivity. *Pain* 36 125–131. 10.1016/0304-3959(89)90120-6 2919090

[B40] Toporikova N. Hagenauer M. H. Ferguson P. Booth V. (2017). “A two-process model for circadian and sleep-dependent modulation of pain sensitivity,” in *Women in Mathematical Biology. Association for Women in Mathematics Series*, Vol. 8 eds Layton A. Miller L. (Cham: Springer).

[B41] Warfield A. E. Prather J. F. Todd W. D. (2021). Systems and circuits linking chronic pain and circadian rhythms. *Front. Neurosci.* 15:705173. 10.3389/fnins.2021.705173 34276301 PMC8284721

[B42] Wen S. Ma D. Zhao M. Xie L. Wu Q. Gou L. (2020). Spatiotemporal single-cell analysis of gene expression in the mouse suprachiasmatic nucleus. *Nat. Neurosci.* 23 456–467. 10.1038/s41593-020-0586-x 32066983

[B43] Wu G. Anafi R. C. Hughes M. E. Kornacker K. Hogenesch J. B. (2016). MetaCycle: an integrated R package to evaluate periodicity in large scale data. *Bioinformatics* 32 3351–3353. 10.1093/bioinformatics/btw405 27378304 PMC5079475

[B44] Zamunér A. R. Barbic F. Dipaola F. Bulgheroni M. Diana A. Atzeni F. (2015). Relationship between sympathetic activity and pain intensity in fibromyalgia. *Clin. Exp. Rheumatol.* 33 S53–S57. 25786044

