# Supplementary figures and images for: The Diurnal Profile of Human Basal Pain Sensitivity and Skin Sympathetic Nerve Activity: A Healthy Volunteer Study
Source: Front Neurosci. 2022 Mar 16;16:810166. doi: 10.3389/fnins.2022.810166 (PMC8966078; doi:10.3389/fnins.2022.810166)

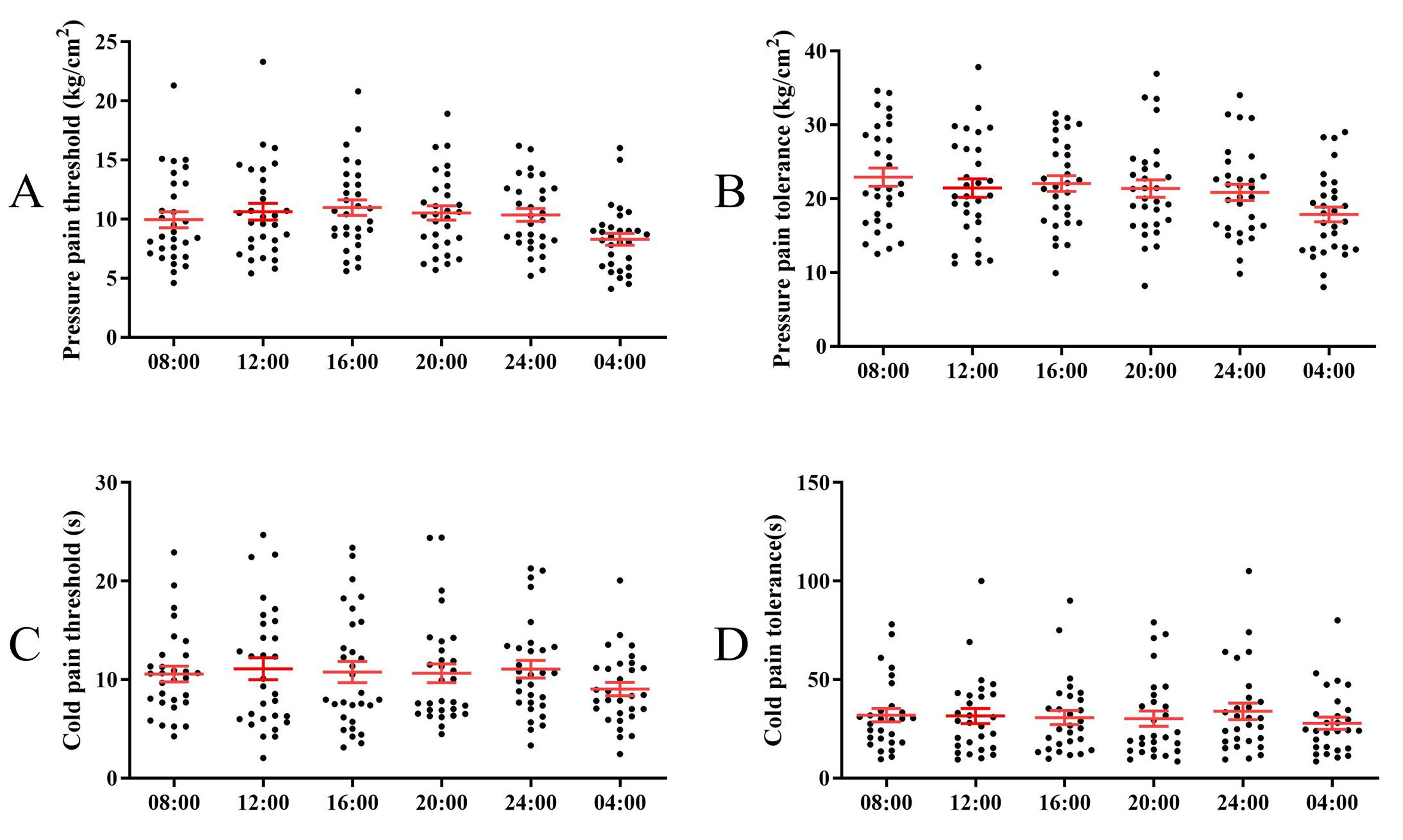

Supplement: Supplementary Figure 1 — The scatter diagrams of the values of pressure pain threshold (A), pressure pain tolerance (B), cold pain threshold (C), and cold pain tolerance (D) at six time points in a day (08:00, 12:00, 16:00, 20:00, 00:00, 04:00). [file Image_1.JPEG]

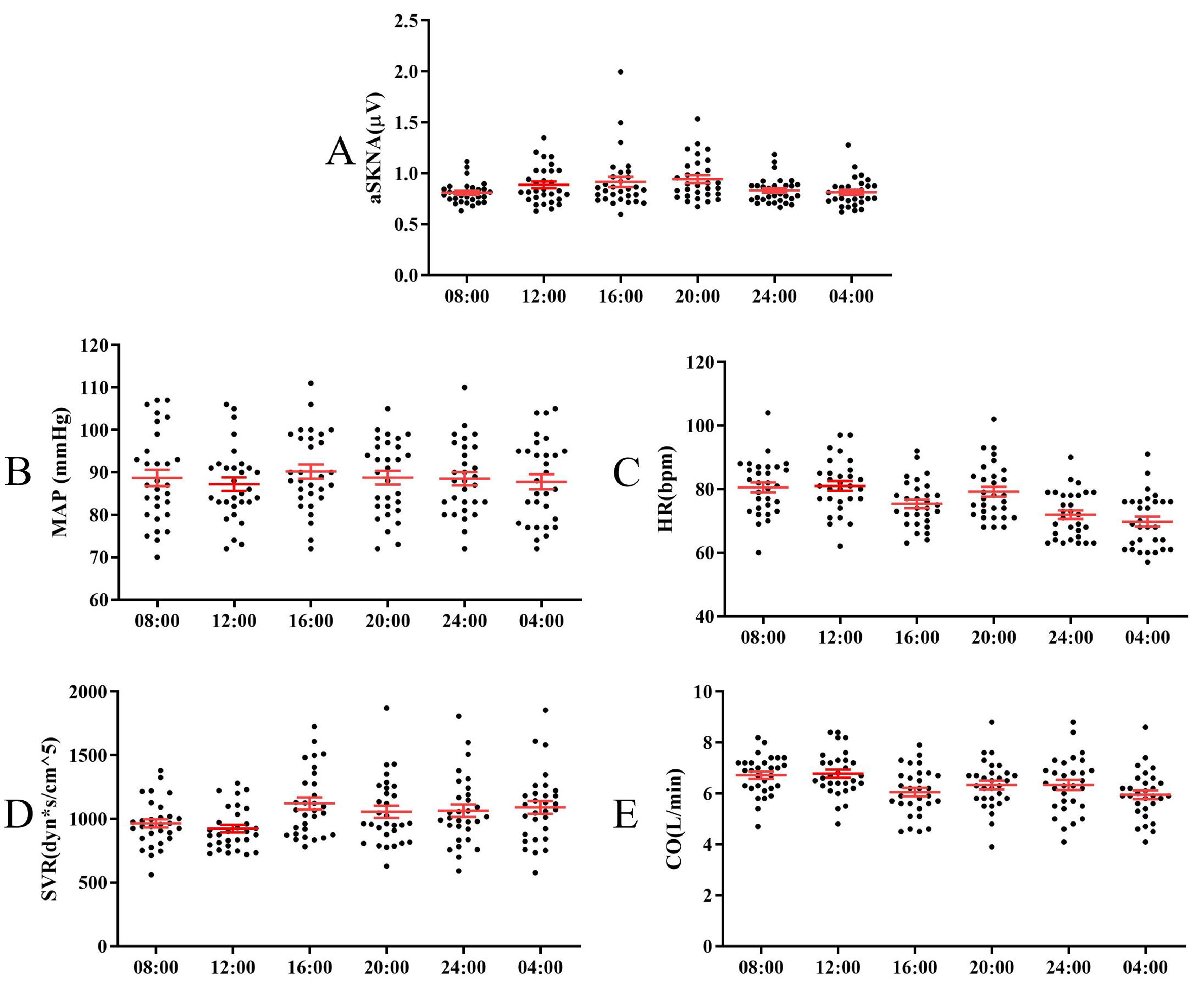

Supplement: Supplementary Figure 2 — The scatter diagrams of the values of average skin sympathetic nerve activity (A), mean arterial pressure (B), heart rate (C), peripheral vascular resistance (D), and cardiac output (E) at six time points of the day (08:00, 12:00, 16:00, 20:00, 00:00, and 04:00). [file Image_2.JPEG]
